# Methodological Challenges in Estimating Trends and Burden of Cardiovascular Disease in Sub-Saharan Africa

**DOI:** 10.1155/2015/921021

**Published:** 2015-12-01

**Authors:** Jacob K. Kariuki, Eileen M. Stuart-Shor, Suzanne G. Leveille, Laura L. Hayman

**Affiliations:** ^1^College of Nursing and Health Sciences, University of Massachusetts, Boston, MA 02125, USA; ^2^Beth Israel Deaconess Medical Center, Boston, MA 02215, USA; ^3^Seed Global Health, 125 Nashua Street, Suite 722, Boston, MA 02114, USA

## Abstract

*Background.* Although 80% of the burden of cardiovascular disease (CVD) is in developing countries, the 2010 global burden of disease (GBD) estimates have been cited to support a premise that sub-Saharan Africa (SSA) is exempt from the CVD epidemic sweeping across developing countries. The widely publicized perspective influences research priorities and resource allocation at a time when secular trends indicate a rapid increase in prevalence of CVD in SSA by 2030.* Purpose.* To explore methodological challenges in estimating trends and burden of CVD in SSA via appraisal of the current CVD statistics and literature.* Methods.* This review was guided by the Critical review methodology described by Grant and Booth. The review traces the origins and evolution of GBD metrics and then explores the methodological limitations inherent in the current GBD statistics. Articles were included based on their conceptual contribution to the existing body of knowledge on the burden of CVD in SSA.* Results/Conclusion.* Cognizant of the methodological challenges discussed, we caution against extrapolation of the global burden of CVD statistics in a way that underrates the actual but uncertain impact of CVD in SSA. We conclude by making a case for optimal but cost-effective surveillance and prevention of CVD in SSA.

## 1. Background

Over the past few decades, researchers have been reporting an epidemiological transition in developing countries, with noncommunicable diseases (NCDs) steadily replacing infectious diseases as the leading cause of morbidity and mortality [1, 2]. CVD, the leading cause of death worldwide, is now responsible for the greatest proportion of NCDs deaths in developing countries [3]. Currently, eighty percent of the global burden of CVD is estimated to be in developing countries where morbidity and deaths occur at younger ages [4].

Although SSA is part of the developing world, many researchers contend that the region is currently exempt from the tsunami of CVD epidemic sweeping the rest of the developing countries [5–7]. The 2010 GBD estimates by the World Health Organization (WHO) have been cited to support the premise that CVD is not a priority public health problem in SSA [7]. This widely publicized perspective influences research priorities and resource allocation for CVD surveillance and prevention at a time when the WHO has projected a rapid increase in prevalence of CVD and other noncommunicable diseases in SSA by 2030.

This paper explores the methodological challenges in estimating trends and burden of CVD in SSA through appraisal of the current CVD statistics and other related literature. Special attention is given to the analysis of assumptions and fundamental limitations inherent in the methods used to derive the global burden of CVD statistics for SSA.

## 2. Methods

The literature review was guided by the Critical review methodology described in the analysis of typology of reviews, by Grant and Booth [8]. Unlike systematic reviews whose hallmark is comprehensiveness of the literature search, a Critical review seeks to explore the conceptual contribution of data from diverse sources to an existing body of knowledge on a subject of interest [8]. Since the GBD statistics are considered as the best available estimates of the burden of CVD in the world [7], this Critical review analyzes and synthesizes data from diverse sources to provide a context for understanding the SSA specific statistics. The review begins by analyzing articles tracing the origins and evolution of GBD metrics and then commences exploring the methodological limitations inherent in the GBD statistics, as implicitly or explicitly acknowledged by the GBD investigators. In line with the Critical review methodology [8], articles were included in this review based on their conceptual contribution to the existing body of knowledge on the burden of CVD in SSA.

## 3. Results/Discussion

### 3.1. Evolution of Metrics Used to Measure Disease Impact on Populations

Prior to the first GBD study in 1990, the health situation of populations was measured primarily using mortality data and other epidemiologic indicators of selected diseases using incidence and prevalence estimates [9]. The progressive decline in incidence and prevalence of fatal infectious diseases, coupled with increasing life spans, shifted interest from a narrow focus on mortality to a broader focus that included functional status and disability. Today, the health situation of populations is assessed through measures of the overall burden of disease. This approach has significantly improved population health assessment by taking into account not only premature deaths but also the morbidities associated with disease and injury [9].

### 3.2. Methods Used to Quantify the Burden of Disease

Summary measures of population health synthesize mortality and morbidity data to represent population health in a single number, enabling standardized comparisons over time and across populations [10]. These measures of population health are generally classified into two broad categories, namely, health expectancies and health gaps [11].

Health expectancies take into account disability and mortality changes which are responsible for increases in life expectancy and help distinguish any tradeoffs between quantity and quality of life [12]. On the other hand, health gaps quantify lost years of full health in comparison with some “ideal” health status or an accepted norm for population health [11]. Both measures are time based estimates in that they multiply the number of years lived or lost to premature mortality by the “quality” of those years.

Approaches to measuring health expectancies include active life expectancy, disability-free life expectancy, disability-adjusted life expectancy, health adjusted life expectancy, and quality adjusted life expectancy. Health gaps indicators include potential years of life lost, healthy years of life lost, quality adjusted life years (QUALYs), and disability-adjusted life years (DALYs) [13]. Detailed discussion of these summary measures of population health is beyond the scope of this paper. However, DALY warrants a brief discussion because it was extensively used in the GBD study to attribute different levels of ill health to various diseases, injuries, and risk factors.

DALY is a two-dimension metric that combines years of life lost due to premature death and years lost due to disability [13]. Each DALY represents a lost year of healthy life, generally calculated by summing up the discounted value of future life years lost due to premature death and the discounted value of future life years adjusted for disability [14]. Taking into account the time lived in disability is particularly important in the 21st Century, which is characterized by a trend of global decline in premature deaths and increasing prevalence of disabling chronic diseases.

Today, the DALYs concept is widely used in estimating the cost-effectiveness of various health interventions, thereby facilitating rational allocation of resources based on the burden of disease [9]. As a general rule, diseases associated with more DALYs should be allocated more resources and given a higher priority in prevention and treatment agenda compared to those accounting for less DALYs.

### 3.3. Statistics on Trends and Burden of CVD in SSA

The high burden of CVD in developing countries has been attributed to progressive increase in prevalence of potentially modifiable CVD risk factors associated with Westernization, coupled with lack of robust preventive interventions [15]. In SSA, the tide of Westernization has been characterized as slower than in other developing countries, therefore explaining why the region is widely regarded as still struggling with preindustrial infectious diseases.

According to the 2010 GBD estimates, CVD accounted for 8.8% of mortalities and 3.52% of all DALYs in SSA [16]. Whereas these statistics seem to support the prevailing premise that CVD is not currently a priority public health problem in SSA, the WHO predictions and other scattered regional data suggest that the dynamics of CVD epidemiology in SSA may not be fully appreciated in the 2010 GBD estimates.

In 2004, a systematic analysis of the GBD by the WHO predicted that by 2030 CVD and other NCDs will surpass communicable, maternal, perinatal, and nutritional diseases as the leading cause of mortality in SSA [17]. These projections further suggested that, during this period, ischemic heart disease will double and become the leading cause of death in Africa if proactive preventive measures are not promptly put into place [6]. Of note, a trend of increasing prevalence of modifiable CVD risk factors has now been reported in several SSA countries that have conducted population based studies on NCDs in the last decade [18].

### 3.4. Methods and Data Sources Used to Derive GBD Statistics

Two methods are generally used to project mortality which is a key component of the DALY. The first method uses aggregate models based on time series analysis of the historical trends of specific and all-cause mortality rates. However, due to their reliance on availability of accurate data on mortality rates, these models are not feasible in many developing countries which lack optimal vital registration records [19].

The second method uses structural models that project mortality as a function of an array of independent factors known to affect the risk of death, such as Gross Domestic Product (GDP), human capital, fertility rate, and smoking intensity [19]. The mortality estimates derived from these models are essentially projections and are preferred when there is limited or unreliable mortality data. The robustness of structural models depends on the extent to which they identify the important determinants of variables of interest and their relationship to mortality rates [19]. The GBD estimates are generally based on the structural modeling approach.

The 2010 GBD statistics are widely referenced as the best available estimates of the burden of CVD in the world [7]. The estimates were based on a wide array of data including vital registration records, verbal autopsy reports, mortality surveillance, censuses, surveys, clinical records, police records, and mortuaries reports [20]. These estimates of the burden of disease are particularly important for developing countries in guiding allocation of resources and in determining cost-effectiveness of various policies and interventions.

### 3.5. Limitations of the Structural Models for SSA

Due to paucity of data on prevalence of CVD risk factors in SSA, the GBD investigators noted that the reported estimates of the burden of CVD in SSA were compromised by low quality data on CVD morbidity and mortality [7]. The data void made the GBD investigators rely more on verbal autopsy reviews, police reports and histories from relatives, mortuary reports, community surveys, and hospital records when estimating the CVD burden in SSA [20].

Prevailing high levels of illiteracy in SSA and the unspecified recall period used by GBD investigators make the validity of the verbal autopsy reports uncertain. Furthermore, in SSA mortuary records and police reports on causes of death are usually inconclusive and unreliable due to various challenges including lack of diagnostic equipment and pathology expertise to investigate cause-specific mortality.

The GBD investigators attempted to mitigate these limitations by introducing into the structural models the established assumptions on the relationships between incidence, prevalence, case fatality, and mortality of specific diseases [7, 21]. However, despite sophisticated statistical maneuvers, the generated statistics for CVD burden in SSA were still marred by a high degree of uncertainty. For instance, the mortality attributable to ischemic heart disease in 2001 was associated with a relative uncertainty of −24% to +34% in SSA compared to about ±12% in developed countries [22].

### 3.6. Assumptions on CVD Epidemiology in SSA

The epidemiological transition theory posits that evolution of mortality in countries has been historically characterized by transition from an era dominated by infectious diseases, high mortality rates, and short life spans to a phase dominated by chronic diseases and injuries, lower mortality rates, and longer life spans. Socioeconomic progress, good public health practices, and technological advances are regarded as important contributing factors to the epidemiological transition process [23].

The classic epidemiologic transition experienced in the West was characterized by CVD epidemic developing first in the affluent social classes, before affecting other social classes including the poor [24, 25]. The use of national income covariates, alongside established assumptions on CVD rates in the GBD models, suggests that the resultant estimates for SSA were modeled according to the classic Western epidemiologic transition pattern.

However, data from the STEPwise approach to Surveillance (STEPS) surveys and other cross-sectional studies in SSA suggest that the Western transition model assumptions may have limited application to the region. The STEPS survey, designed by the WHO in 2000, collects data using three steps. Step one captures demographics and biobehavioral risk factors such as tobacco use, alcohol consumption, physical inactivity, and fruit and vegetable consumption. Step two includes physical measurements of weight, height, waist circumference, and blood pressure, while step three captures biochemical measures such as fasting blood sugar and total cholesterol [18]. These risk factors are associated with CVD and other major chronic diseases.

To date, the STEPS surveys have been done in more than a dozen SSA countries using standardized questions and protocols, thus enabling comparison of risk factor distributions across all participating countries. Although the data generated so far depicts significant heterogeneity within and between countries, more than 75% of all STEPS participants in SSA have had at least one major risk factor for CVD. The most prevalent risk factors observed across the region include high age-adjusted BMI especially in women, elevated systolic blood pressure, low consumption of fruits and vegetables, and increased levels of fasting blood glucose [5].

A Malawian national representative survey conducted in 2009 using the STEPS approach reported that the age-adjusted prevalence of hypertension was 33.2% in participants aged between 25 and 64 years. Seventy-five percent of these participants reported never having their blood pressure checked previously, and over 94.9% of those with hypertension were not aware of their condition [26]. Similar observations have been made by other researchers in SSA who have reported high rates of hypertension, sometimes exceeding those observed for the same age group in developed countries [27]. These data allude to a possible deviation from the classic Western epidemiologic transition model and a unique pattern of rising prevalence of CVD risk factors in the absence of significant Westernization.

### 3.7. Lessons from Developed Countries

Developed countries provide time tested case studies from which countries in the SSA region could borrow important lessons. The foremost lesson is the public health and socioeconomic ramifications of a high burden of CVD. Despite having strong healthcare systems, advanced medical technologies, and abundance of resources, the developed countries have been dominated by high CVD mortality since the advent of the 20th Century. In 2010, the United States spent approximately $503.2 billion to manage CVD. Despite the staggering healthcare expenditure, it has been estimated that about 2300 Americans die each day of CVD related causes [28].

The enormous cost of CVD should be viewed as a warning that a CVD epidemic might create an enormous burden in SSA. It is obvious that the region lacks the requisite healthcare infrastructure and resources to handle the direct and indirect implications of a full blown CVD epidemic. Another important lesson is the effectiveness of integrated and well-designed CVD surveillance and management strategies leading to a steady decline in age-adjusted mortality [15]. To optimize and integrate risk surveillance with prevention, many developed countries have adopted proactive screening strategies using the absolute CVD risk approach that allows approximation of the likelihood that a particular set of risk factors will lead to CVD related morbidity or mortality within a given time frame [29].

Clinical guidelines in most developed countries have adopted the absolute CVD risk approach to maximize opportunities for early detection and prompt treatment of subclinical CVD risk in their populations [30, 31]. In addition, data that enable calculation of absolute CVD risk are routinely collected in the ongoing population based studies in these countries. These data are used to stratify CVD risk in the study population, and results are extrapolated to establish countrywide risk profiles and cost-effective thresholds for various levels of treatment [32].

### 3.8. A Case for Optimal CVD Surveillance and Prevention in SSA

The most prudent conclusion is that the actual burden and trend of CVD in SSA is not fully understood. Nevertheless, there is consensus on the unmistakable trend of increasing prevalence of CVD risk factors in the region. Unfortunately, policymakers and other key stakeholders in the region appear to be making decisions based on a flawed extrapolation of the 2010 GBD statistics that the DALYs associated with CVD are too few for CVD to be given a priority in the region's public health agenda.

The World Bank budgetary estimates suggest that the current health policies in SSA are not geared to address the emerging CVD epidemic in the region. For more than 10 years, the World Bank reports indicate that countries in SSA have been allocating less than 10% of their GDP to healthcare. Only about 20% of these restricted budgets have been allocated for all noncommunicable diseases during this period [33]. The same trend is observed with the WHO which allocated only 12% of its 2008-2009 budget for all noncommunicable diseases [34]. Continuation of this skewed allocation of resources could potentially lead to missed opportunities for mapping the actual burden of CVD and implementation of effective surveillance and prevention strategies.

It has been estimated that, with effective surveillance and treatment strategies, CVD mortality could be reduced by up to 50% in developing countries [35]. In SSA where resources are most scarce, researchers have demonstrated impressive benefits of proactive prevention and treatment efforts compared to no interventions. For instance, primary prevention efforts targeting populations with more than 25% ten-year absolute risk of CVD were associated with an incremental cost-effectiveness ratio of $771 for each healthy year of life saved (QALY) [35].

Despite the current economic constraints in SSA, the estimated cost-effectiveness ratios for primary prevention are still considered feasible because they are below the WHO threshold which considers an intervention to be cost-effective if it costs less than three times the gross national income per head to gain a QALY [36].

### 3.9. Pragmatic Steps towards Surveillance and Prevention of CVD in SSA

Population based studies have clearly demonstrated that the main pathological pathways leading to CVD begin early in life and progress cumulatively through adolescence and adulthood [37]. Although each major CVD risk factor independently increases the likelihood of CVD events in exposed individuals, clustering of multiple risk factors is known to compound the CVD risk [31]. Therefore, focusing on individual risk factors, as is the current practice in many SSA clinical settings and community based surveys, may under/overrate risk in populations.

Integrating absolute CVD risk assessment into routine clinical assessment and in the ongoing population based surveys will foster standardized opportunistic and proactive CVD risk surveillance in the region. Due to lack of locally derived alternatives, existing algorithms developed in developed countries provide a beginning point for initiating efficient CVD surveillance and prevention systems in SSA. Adoption of algorithms developed in significantly different cohorts is recommended under the assumption that the major risk factors for CVD are fairly similar around the world. The INTERHEART investigators identified 9 major risk factors (smoking, lipids, hypertension, diabetes, obesity, unhealthy diet, physical inactivity, harmful alcohol consumption, and psychosocial factors), which account for over 90% of the population attributable risk of acute myocardial infarction worldwide [38].

The feasibility of adopting these algorithms in SSA has been elusive because they were primarily based on laboratory measures which are generally inaccessible in the region. The development of non-laboratory based algorithms in recent years provides an opportunity for initiating opportunistic and proactive screening programs that will capture absolute risk in SSA. It has been estimated that an individual's absolute risk score can be calculated within five to ten minutes using these algorithms because the only data required to estimate absolute risk include age, BMI, systolic blood pressure, antihypertensive medication use, current smoking, and diabetes status [39, 40].

Since the equipment and level of expertise needed to assess absolute risk using these non-laboratory based algorithms are readily available in SSA, absolute CVD risk assessment can be incorporated with ease into routine clinical assessments and also into ongoing health surveys in SSA. This initiative would require minimal investments in training local clinicians and data collectors on how to use the algorithms and the practical applications of the absolute risk scores. Opportunistic and proactive screening initiatives guided by the absolute risk approach will incrementally pool quality data on population-specific CVD risk profiles. Such data is likely to help countries in making sound decisions on the need for primary or secondary prevention interventions and can help improve the future structural models for estimating the burden of CVD in SSA.

## 4. Conclusion

Cognizant of the methodological challenges discussed in this paper, we contend that instead of minimizing the significance of CVD in SSA based on the current GBD estimates efforts should be channeled towards more research and in establishing efficient risk surveillance and preventive systems. Such systems, guided by the absolute CVD risk approach, could begin by examining traditional risk factors, such as those included in the non-laboratory based CVD risk assessment algorithms [39, 40]. Eventually, the surveillance systems can be adapted to include novel trends and population-specific risks identified through local data and CVD patterns.
